# Down-Regulation of Neogenin Accelerated Glioma Progression through Promoter Methylation and Its Overexpression in SHG-44 Induced Apoptosis

**DOI:** 10.1371/journal.pone.0038074

**Published:** 2012-05-29

**Authors:** Xinmin Wu, Yunqian Li, Xilin Wan, Tabitha Mlowoka Kayira, Rangjuan Cao, Xingda Ju, Xiaojuan Zhu, Gang Zhao

**Affiliations:** 1 Key Laboratory of Molecular Epigenetics of Ministry of Education, Institute of Cytology and Genetics, Northeast Normal University, Changchun, China; 2 Department of Neurosurgery, the First Hospital of Jilin University, Changchun, China; University of Chicago, United States of America

## Abstract

**Background:**

Dependence receptors have been proved to act as tumor suppressors in tumorigenesis. Neogenin, a DCC homologue, well known for its fundamental role in axon guidance and cellular differentiation, is also a dependence receptor functioning to control apoptosis. However, loss of neogenin has been reported in several kinds of cancers, but its role in glioma remains to be further investigated.

**Methodology/Principal Findings:**

Western blot analysis showed that neogenin level was lower in glioma tissues than in their matching surrounding non-neoplastic tissues (n = 13, *p*<0.01). By immunohistochemical analysis of 69 primary and 16 paired initial and recurrent glioma sections, we found that the loss of neogenin did not only correlate negatively with glioma malignancy (n = 69, *p*<0.01), but also glioma recurrence (n = 16, *p*<0.05). Kaplan-Meier plot and Cox proportional hazards modelling showed that over-expressive neogenin could prolong the tumor latency (n = 69, *p*<0.001, 1187.6±162.6 days versus 687.4±254.2 days) and restrain high-grade glioma development (n = 69, *p*<0.01, HR: 0.264, 95% CI: 0.102 to 0.687). By Methylation specific polymerase chain reaction (MSP), we reported that neogenin promoter was methylated in 31.0% (9/29) gliomas, but absent in 3 kinds of glioma cell lines. Interestingly, the prevalence of methylation in high-grade gliomas was higher than low-grade gliomas and non-neoplastic brain tissues (n = 33, *p*<0.05) and overall methylation rate increased as glioma malignancy advanced. Furthermore, when cells were over-expressed by neogenin, the apoptotic rate in SHG-44 was increased to 39.7% compared with 8.1% in the blank control (*p<*0.01) and 9.3% in the negative control (*p*<0.01).

**Conclusions/Significance:**

These observations recapitulated the proposed role of neogenin as a tumor suppressor in gliomas and we suggest its down-regulation owing to promoter methylation is a selective advantage for glioma genesis, progression and recurrence. Furthermore, the induction of apoptosis in SHG-44 cells after overexpression of neogenin, indicated that neogenin could be a novel target for glioma therapy.

## Introduction

Gliomas are the commonest primary tumors in central nervous system (CNS) arising from neuroepithelial cells and they account for over 50% of primary brain tumors. Recent epidemiological reports showed that the incidence of gliomas was about 6.5/100,000 and the mortality was about 4.3/100,000 per year and the 5-year survival rate was only 33.4% [1], [2]. In clinical pathological classification, gliomas are categorised as astrocytoma, oligodendroglioma, oligoastrocytoma, glioblastoma among others. Depending on pathological characteristics of tumor, gliomas are divided into four grades: I, II, III and IV in ascending order of malignancy. Generally, grade I and II are considered as low-grade gliomas while grade III and IV are high-grade gliomas [3]. The pattern for growth of high-grade glioma is infiltrating and dilative, with the invasion and destruction of surrounding tissues. So far, surgery is the main therapeutic regimen but microscopic glioblastoma, also described as Grade IV glioma, often infiltrates much larger areas than is seen on Magnetic Resonance Imaging (MRI) or Computed Tomography (CT), and as such surgical treatment is at best insufficient, consequently post-operative recurrence is frequently encountered accompanied by short survival time of approximately 14 months [4]. Globally, developed countries registered a higher mortality rate than developing countries in primary gliomas [5]. Current research has reported that ionising radiation, chemical and genetic susceptibility are main etiologic factors in glioma formation [4]. However, molecular mechanism for glioma progression and recurrence is still not precisely understood. There is anticipation that integration of genetic and/or molecular information would not only assist pathologists in differentiating glioma grades and disease progression rate, but also uncover molecular markers that could aid in diagnosis and prognosis.

Neogenin, a homologue of Deleted in Colorectal Cancer (DCC), is widely distributed in the CNS [6]–[8] as a dependent receptor of repulsive guidance molecule a (RGMa). It was first identified after separation from chicken embryo where it was reported to regulate end stage of cellular differentiation [9]. Subsequent studies revealed its roles in cell migration and development, apoptosis, histogenesis, angiogenesis and epithelial cell renewal [10]–[14]. It belongs to immunoglobulin super family consisting of four immune globulins followed by six fibronectin type III domains in the extracellular region, a transmembrane region and a cytoplasmic region endowed with death domain [15]. It is reported that alteration of expression in the dependent receptors such as DCC, UNC5H, neogenin [16] and their ligands, netrin-1 [17] and RGMa [18], [19] would cause loss of pro-apoptotic activity and even lead to tumorigenesis [20]–[22]. In recent years, dependent receptors have been identified as potential cancer inhibitors in various cancers [23]–[25]. Gain of netrin-1 and/or loss of DCC would accelerate glioma progression [26]–[29]. Down-regulation of neogenin has been reported in primary breast cancer and metastatic breast cancer relative to the normal breast tissues [30]. Berrar and colleagues demonstrated that in lung cancer patients, shorter survival time was exhibited in those with lower expression of neogenin compared with their counterparts with higher expression [31]. Though neogenin is widely distributed in CNS, its role in gliomas remains to be elucidated. In this study we probed the correlation by immunological, promoter methylation and flow cytometry techniques.

## Results

### Lower Expression of Neogenin in Gliomas than Surrounding Areas

**Figure 1:**
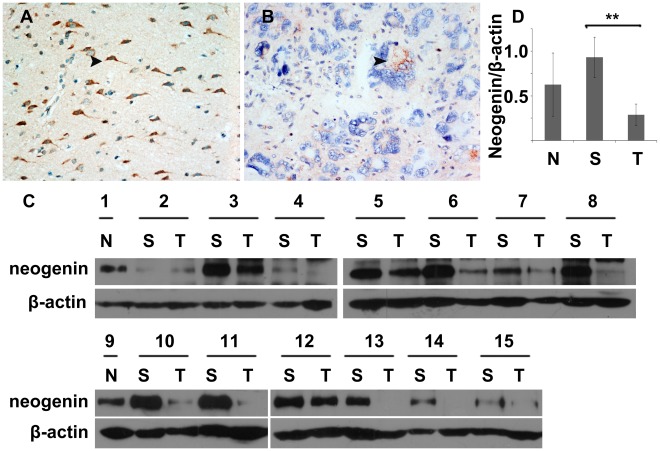
The difference of neogenin expression between gliomas and their homologous surrounding tissues. (A–B) Representative immunohistochemical pictures (200× magnification): (A) The surrounding tissue of the tumor, the arrow points to a positive normal cell; (B) The typical glioblastoma area, the arrow points to positive tumor cell. (C) Western blot showed neogenin expression in normal brains (N), surrounding non-neoplastic tissues (S) and homologous gliomas (T). (D) Statistical graph of neogenin expression in surrounding non-neoplastic tissues and gliomas. Paired *t* -test, n = 13, ** *p*<0.01, error bars indicate standard error means.

In this study, a clear pattern of neogenin expression in gliomas was the first priority. Two normal brain tissues and thirteen paired surrounding and glioma tissues (Table S1) were analysed (each glioma tissue was paired with its neighbouring non-neoplastic tissue). Immunohistochemistry results showed that cells in the surrounding tissues were sparse, well distributed, rarely polynuclear and karyokinetic, normal glial cell shape though seldom hyperplasia (Fig. 1A), whereas in the typical tumor tissues, cells were more heterogenous: ranging from small to giant, disordered, polynuclear to karyokinetic (Fig. 1B). Western blot analysis showed that neogenin in tumor tissues was lower relative to that in the normal brain tissues and surrounding non-neoplastic tissues, using β-actin as an internal control for electrophoresis efficiency (paired *t*-test, n = 13, *p*<0.01, Fig. 1C, D). Strikingly, the down-expression of neogenin was more clear in high-grade gliomas (n = 7) and female patients (n = 7) than low-grade gliomas and male patients (Table 1, paired t-test, n = 7, *p*<0.01). These results signified that the expression of neogenin was down-regulated in gliomas and more pronounced in high-grade gliomas.

**Table 1:** Clinicopathologic features and relative neogenin level profile in 13 paired surrounding and glioma tissues (January – November, 2011).

			Ratio of neogenin/β-actin(Mean±SD)		
Feature	Variable	No.(%)	Surrounding	Tumor	*p*-value*
Gender	Female	7(53.8)	0.734±0.426	0.110±0.208	0.005
	Male	6(46.2)	1.159±1.126	0.498±0.550	0.079
Histology	Low-grade	6(46.2)	0.688±0.484	0.290±0.339	0.336
	High-grade	7(53.8)	1.137±1.015	0.288±0.530	0.004
Total	Glioma	13(100)	0.930±0.817	0.289±0.434	0.001

SD: standard deviation;

* : paired *t*-test.

### Negative Relationship between the Expression of Neogenin and Glioma Malignancy

**Figure 2:**
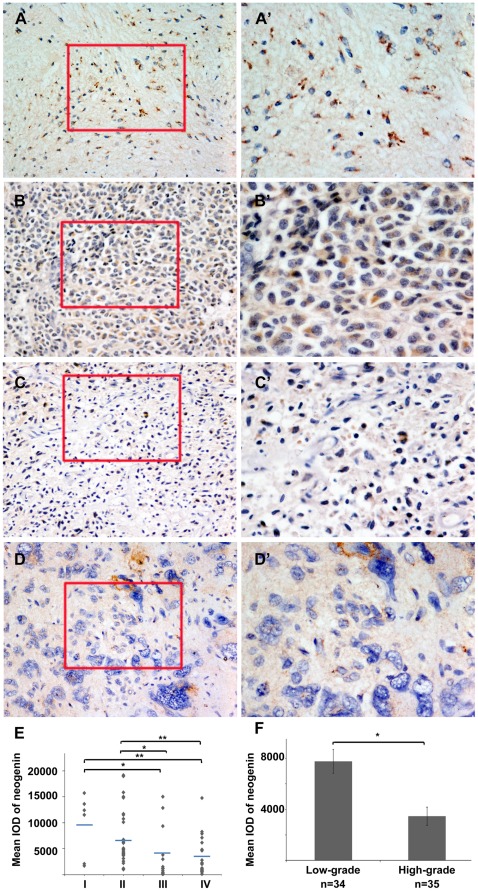
Immunohistochemical analysis of neogenin in primary gliomas. (A–D) Representative immunohistochemical pictures of grade I-IV glioma respectively, (A’–D’) Magnification of the red squares in (A–D), (A–D) 200× magnification; (A’–D’) 400× magnification. (E) Scatter diagram of neogenin expression in 69 primary gliomas, blue bars are the means of four grades, n = 69, One-Way ANOVA was used in statistical analysis. (F) Histogram of mean neogenin expression in low-grade gliomas and high-grade gliomas, statistical analysis was performed with independent *t*-test. *p<0.05, **p<0.01, error bars indicate standard error means.

Since there was loss of neogenin in glioma tissues compared to their surrounding non-neoplastic tissues, sixty nine primary glioma cases (Table S2) from January, 2006 to January, 2011 in the Pathological Specimen Library of the First Clinical Hospital of Jilin University were collected for examination of relationship between neogenin expression and glioma malignancy. All sections were diagnosed as gliomas according to pathological characteristics and classified into grade I-IV: including grade I (n = 6), grade II (n = 28), grade III (n = 14) and IV (n = 21) respectively (Table 2). The results from immunohistochemical assay showed that expression of neogenin was decreased, which was associated with grade advancement (Fig. 2A–D and A’–D’). The average integral optical density (IOD) of positive signal representing the relative neogenin level was significantly different between grade I and III, grade I and IV, grade II and III, and grade II and IV (One-Way ANOVA, n = 69, *p*<0.01, Fig. 2E), and also was distinct between the low-grade and the high-grade gliomas (independent *t*-test, n = 69, *p*<0.05, Fig. 2F, Table 2). These results indicated that down-regulation of neogenin was negatively correlated with the grade advancement which meant that malignancy of gliomas was promoted.

**Table 2:** The 69 patients’ classified statistic according to clinical and pathological features (January, 2006 – January, 2011).

		Grade I		Grade II		Grade III		Grade IV		Total		
Feature	Variable	No.(%)	IOD	No.(%)	IOD	No.(%)	IOD	No.(%)	IOD	No.(%)	IOD	*p*-value
Gender	Female	3(4.3)	5805±6771	9(13.0)	8476±6537	9(13.0)	1468±1179	10(14.5)	2373±2896	31 (44.9)	4214±5141	0.309*
	Male	3(4.3)	13201±2217	19(27.5)	6851±4734	5(7.2)	7511±6992	11(15.9)	4237±4253	38 (55.1)	6683±5164	–
Age	≤30 years	6(8.7)	9503±6059	9(13.0)	7327±5583	1(1.4)	48	5(7.2)	1971±1840	21 (30.4)	6327±5702	0.424†
	31–60 years	0	–	18(26.1)	7602±5409	12(17.4)	4189±5199	13(18.8)	3861±4349	43 (62.3)	5519±52478	–
	>60 years	0	–	1(1.4)	3673	1(1.4)	449	3(4.3)	3428±3248	5 (7.2)	2881±2671	–
Latency	≤3 months	2(2.9)	13016±854	13(18.8)	5619±4226	8(11.6)	2613±5045	16(23.2)	2375±2453	39 (56.5)	4051±4406	0.039*
	>3 months	4(5.8)	7746±6972	15(21.7)	8894±5808	6(8.7)	4977±5034	5(7.2)	6468±5488	30 (43.5)	7553±5688	–
In-hospital	≤20 days	5(7.2)	8679±6388	22(31.9)	7158±4889	11(15.9)	3103±4180	15(21.7)	3322±4110	53 (76.8)	5374±5040	0.309*
	>20 days	1(1.4)	13620	6(8.7)	8164±7136	3(4.3)	5545±8226	6(8.7)	3418±2758	16 (23.2)	6234±6077	–
Location	Supratentorial	2 (2.9)	7252±7298	26(37.7)	7365±5442	14(20.3)	3626±4993	21(30.4)	3349±3705	63 (91.3)	5192±5126	0.785*
	Subtentorial	4(5.8)	10628±6195	2 (2.9)	7484±4582	0	–	0	–	6 (8.7)	9580±5464	–
Size	≤3.00 cm	2(3.5)	12553±1509	4 (7.0)	5706±3741	1(1.8)	3785.6	1(1.8)	864.7	8 (14.0)	6573±4768	0.835†, ‡
	3.01–6.00cm	3(5.3)	9940±7321	16(28.1)	8038±5812	7(12.3)	2809±5409	14(24.6)	3626±3933	40 (70.2)	5722±5663	–
	>6.00 cm	1(1.8)	2092	3(5.3)	8161±6664	3(5.3)	4488±7218	2(3.5)	4596±3594	9 (15.8)	5470±5517	–
Histology	Low-grade	6(8.7)	9503±6059	28(40.6)	7373±5310	0	–	0	–	34(49.3)	7749±5414	0.018*
	High-grade	0	–	0	–	14(20.3)	3626±4993	21(30.4)	3349±3705	35(50.7)	3460±4198	–
Total	Glioma	6(8.7)	9503±6059	28(40.6)	7373±5310	14(20.3)	3626±4993	21(30.4)	3349±3705	69(100)	5574±5263	0.005†

IOD: integral optical density;

* : independent *t*-test,

† : One-Way ANOVA. All tumor sizes were measured by CT or MRI except the data from visual inspection in the operation (details in Table S2),

‡ : n = 57. IOD values were supplied as Mean±Standard Deviation.

### Down-regulation of Neogenin Expression Accelerated the Progress of Gliomas

**Figure 3:**
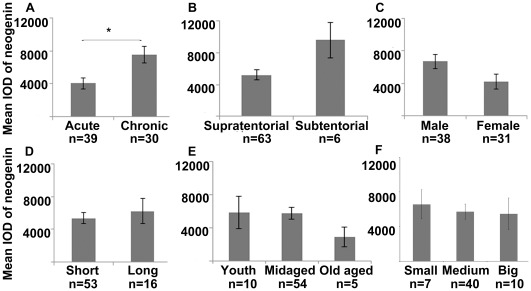
The classified statistics of neogenin expression in 69 primary gliomas. (A–D) Histograms of mean neogenin levels in different statistic groups: (A) tumor latency, (B) tumor location, (C) gender, (D) duration of being in hospital, (E) age, (F) tumor size. N, the number of cases in different statistic groups, statistical methods were performed with independent *t*-test and One-Way ANOVA, *p<0.05, error bars indicate standard error means.

Then, we wondered whether the expression level of neogenin was also correlated with clinical characteristics of gliomas. The clinical features of 69 primary patients used above were analysed, including tumor latency (the period from the first symptom to diagnosis [32], [33]), age, gender, tumor size and location (Table 2 and Table S2). Remarkably, neogenin expression was lower in acute latency group, indicating that its loss may accelerate glioma progress (Fig. 3A, independent *t*-test, n = 69, *p*<0.05). However, there was no statistical difference of neogenin expression between tumor location, gender, length of being in hospital, age and tumor size (Fig. 3B–F).

**Figure 4:**
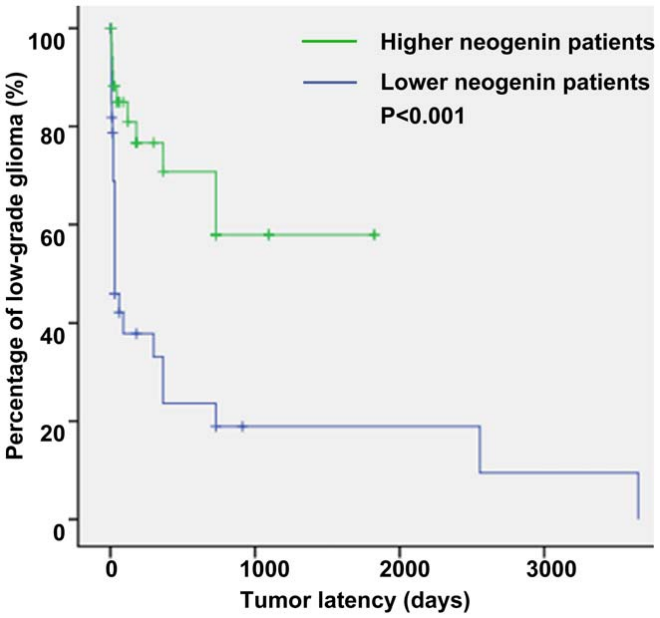
Kaplan-Meier survival curves for overall progression to high-grade glioma. Failure event for computation of this curve was diagnosed as the high-grade glioma. Higher neogenin patients are marked in green color, lower neogenin patients are marked in blue color.

Furthermore, Kaplan-Meier curve and Cox proportional hazards modelling were used to identify the potential relationship between neogenin and high-grade gliomas. The high-grade gliomas were defined as the Failure event. The patients (n = 69) were divided into two groups by Median, the middle number of IOD in the list (Table S2). The Kaplan-Meier plot showed that the patients with overexpression of neogenin had more low-grade glioma rate (71.4% *versus* 26.5%) and longer tumor latency (1187.6±162.6 days *versus* 687.4±254.2 days) (n = 69, *p*<0.001, Fig. 4). Multivariable analysis demonstrated that neogenin could be a beneficial factor to restrain low-grade gliomas from progressing into higher more aggressive grades that are renowned for poor clinical outcome (n = 69, *p*<0.01, Hazard Ratio 0.264, 95% Confidence Interval: 0.102 to 0.687, Table 3).

**Table 3:** Multivariate hazard ratios of progression to high-grade gliomas in 69 primary glioma patients (January, 2006 – January, 2011).

			95.0% Confidence Interval	
Factor	*p*-value	Hazard Ratio	Lower	Upper
Neogenin (Higher expression vs. Lower expression) ^‡^	0.006	0.264	0.102	0.687
Tumor size (Increase of 3 cm)	0.809	1.077	0.592	1.960
Gender (Males vs. Females)	0.439	1.424	0.582	3.489
Age (Increase of 30 years)	0.422	1.333	0.661	2.689

Multivariate hazard ratios and 95% Confidence Intervals were obtained by using Latency as time-scale and High-grade glioma as Failure event. ^‡^: divided by Median of neogenin IODs listed in the Table S2.

### Down-regulation of Neogenin Accelerated the Recurrence of Gliomas

**Figure 5:**
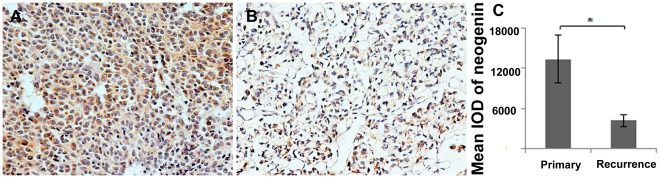
Expression of neogenin in 16 paired primary and recurrent glioma sections. (A-B) Representative immunohistochemical pictures (200× magnification): (A) primary glioma, (B) matching recurrent glioma. (C) statistical graph of mean neogenin expression in primary and recurrent gliomas. Paired t-test, n = 16, * *p*<0.05, error bars indicate standard error means.

In order to examine the association between neogenin and glioma recurrence, paired primary and recurrent sections (n = 16, Table S3) were collected from 16 patients, who were diagnosed as gliomas during January 2001 to January 2011. The result showed that mean expression of neogenin in primary tissues was significantly higher than the recurrent ones (Fig. 5A–C, Table 4 and Table S3, paired *t* -test, n = 16, *p*<0.05). Interestingly, the down-regulation in low-grade gliomas (n = 5, *p*<0.05) and male patients (n = 11, *p*<0.05) was more significant than high-grade gliomas and female patients in the recurrent cases (Table 4). These results may offer a clue that progressive down-regulation of neogenin could be one of the factors behind glioma reappearance after resection.

**Table 4:** Primary and recurrent clinicopathologic features of 16 patients (January, 2001 – January, 2011).

Feature	Variable	No.(%)	PrimaryIOD	RecurrentIOD	*p*-value*
Gender	Female	5(31.3)	5631±7750	4002±4473	0.745
	Male	11(68.8)	16905±15683	4217±3552	0.015
Histology	Low-grade	5(31.3)	13397±7798	2687±2057	0.025
	High-grade	9(56.3)	11387±16083	4824±4626	0.226
	Uncertain†	2(12.5)	22317±25073	4772±1635	–
Total	Glioma	16(100)	13381±14461	4149±3709	0.018

IOD: integral optical density;

IOD values were supplied as Mean±Standard Deviation.

* : paired *t*-test;

† : undefined grade gliomas.

### Neogenin Promoter was Methylated in Gliomas

**Figure 6:**
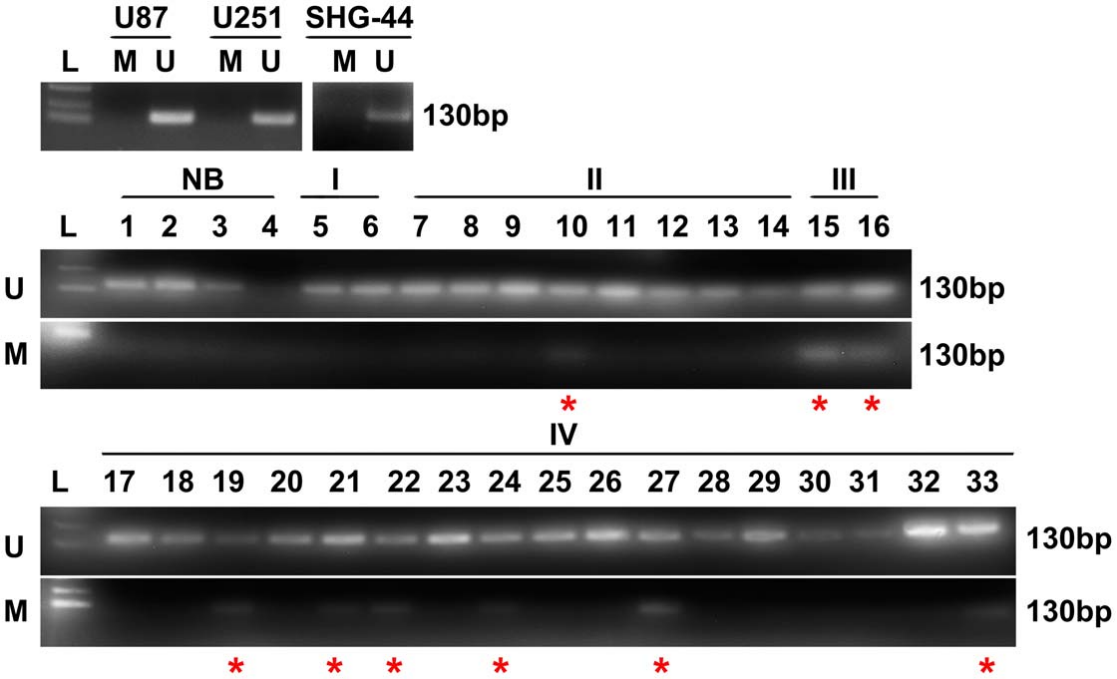
Methylation of neogenin promoter in 3 kinds of glioma cell lines, 4 non-neoplastic brain tissues and 29 gliomas. L: ladder, NB: non-neoplastic brain tissues, U: unmethylated, M: methylated, red asterisks indicate methylation.

Accumulating evidences indicate that cancer is the result of various genetic and epigenetic alterations of tumor suppressor genes [34]. It has been reported in gliomas, isocitrate dehydrogenase mutation 1 (IDH1) is associated with DNA methylation phenotype [35]. Furthermore, data from microarray studies also showed existence of a glioma-CpG Island methylator phenotype (G-CIMP) that was used to define a distinct subgroup of glioma [36]. The profile of somatic epigenetic as well as genetic alteration is central to understand the pattern of disrupted cellular function responsible for deadly behaviour of gliomas. We selected methylation as an epigenetic marker which has been established in most cancers as one of the reasons responsible for gene deregulation. By analysing the sequence of neogenin promoter, we found that there were two CpG islands prone to methylation. Subsequent methylation-specific polymerase chain reaction (MSP) on the neogenin promoter validated the presence of epigenetic alteration in glioma. In agreement with our hypothesis, 31.0% of gliomas (9/29) were methylated, while no methylation was observed in the non-neoplastic brain tissues (0/4) and cell lines U87MG, U251MG and SHG-44 (0/3). Intriguingly, it was observed that 12.5% (1/8) of grade II, all of grade III (2/2) and 35.3% (6/17) of grade IV glioma were methylated but no methylation in grade I (0/2). Combining above data, 42.1% (8/19) of high-grade gliomas presented methylation of neogenin promoter, compared with 10% (1/10) of low-grade gliomas. Classified statistic showed that the methylation in high-grade gliomas was more frequent than low-grade gliomas and non-neoplastic brain tissues (Fig. 6, Table 5, Chi-square test, n = 33, *p*<0.05), indicating that the ratio of methylation gradually increased with glioma grade.

**Table 5:** Clinicopathologic features and the methylation of neogenin in 4 non-neoplastic brain tissues and 29 glioma tissues (January – November, 2011).

Feature	Variable	No. (%)	Methylated no. (%)	Unmethylated no. (%)	Methylation Rate(%)	*p*-value*
Gender	Female	17(51.5)	3(9.1)	14(42.4)	17.6	0.198
	Male	16(48.5)	6(18.2)	10(30.3)	37.5	–
Age	≤60	27(81.8)	6(18.2)	21(63.6)	22.2	0.186
	>60	6(18.2)	3(9.1)	3(9.1)	50.0	–
Histology	NB	4(42.4)	0(0)	4(12.1)	0	0.018†
	Low-grade	10(30.3)	1(3.0)	9(27.3)	10.0	–
	High-grade	19(57.6)	8(24.2)	11(33.3)	42.1	–
Total	Glioma	29(87.9)	9(27.3)	20(60.6)	31.0	–

NB: non-neoplastic brains;

* : Chi-square test;

† : the compare between the sum of NB and low-grade gliomas and high-grade gliomas.

### Induction of Apoptosis after Neogenin Overexpression in SHG-44 Cell Line

**Figure 7:**
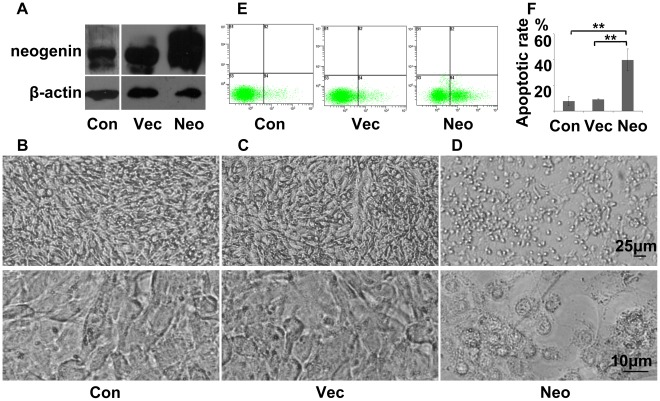
Overexpression of neogenin in SHG-44 cell line. (A) Western blot results showed neogenin expression in the blank control which was untreated (Con), the negative control which was transfected by empty vector (Vec) and the over-expressive group which was transfected by neogenin expression plasmid (Neo) in SHG-44 cell line at 48 hours after transfection; (B-D) Status of cells in the blank, negative and over-expressive group of neogenin respectively at 48 h after transfection. (E) Apoptotic distribution maps of cells in the blank, negative and over-expressive group were drew by flow cytometry assay respectively. (F) Average apoptotic rate of cells in the blanck, negative and over-expressive group in the flow cytometry assay, One-Way ANOVA, n = 3, **p<0.01, error bars indicate standard error means.

Then, to further understand the role of neogenin in glioma, it was over-expressed in cell line SHG-44 by transfection. Cells were harvested at 48 hours and analysed by Western blot for verification of neogenin expression (Fig. 7A). During the cell culture, we found that cells with over-expressive neogenin dissociated from each other unlike in blank and negative control, in which cells grew in cohesion (Fig. 7B–D). In addition, flow cytometry assay showed that 39.7% of cells transfected with neogenin underwent apoptosis compared to 8.1% in the blank control (*p<*0.01) and 9.3% in the negative control, which were not transfected and transfected with empty vector respectively (*p<*0.01, Fig. 7E and F). These observations further indicated that neogenin was a glioma suppressor by inducing apoptosis in SHG-44 cells.

## Discussion

Contrary to the observations made by Meyerhardt and co-workers in 1997 [37], where they reported that neogenin was unaffected in a range of cancers including glioblastoma, the conclusions from Hanninen et al showed that the neogenin mRNA levels were lower in oligodendrogliomas, oligoastrocytomas, medulloblastomas and astrocytomas than in normal brain regions [38]. Moreover, down-regulation of neogenin expression was also reported in colon cancers [39], prostate tumors [40] and breast cancers [30]. Our results supported that neogenin expression in the typical glioma areas was lower than in the matching surrounding areas (n = 13). In agreement with expression of other dependent receptors like DCC and UNC5H which have been regarded as tumor suppressors because of their reported loss of function in variety of cancers and pro-apoptotic ability [41], [42]. Taken together, neogenin is shown to play the role of tumor suppressor in glioma.

The interesting finding in this study was that neogenin expression was negatively related with glioma grade (n = 69). Down-regulation of neogenin expression could increase the degree of malignancy. Importantly, in most of the recurrent cases, neogenin expression was further reduced compared with the matching primary tumors from the same patient especially in the low-grade gliomas, indicating that progressive neogenin loss in glioma might begin at early stage and promote its recurrence after resection. As expected, the phenotype for loss of neogenin expression could be similar to DCC, inactivation of which helped to further glioma progression and glioblastoma regeneration [27], [43].

On the basis of clinical experiences, malignancy of glioma is closely correlated with tumor latency, size, occasionally with patient’s age among other factors. The patients with acute tumor latency probably have higher risk of developing high-grade glioma. By exploring the correlation between neogenin and glioma latency, we found that expression of neogenin was lesser in gliomas with acute latency, meaning that over-expressive patients were with lower hazard ratio to advance to high-grade gliomas. Epidemiological study suggests that males are more prone to gliomas than females [2]. But depending on neogenin expression, loss of neogenin was more pronounced in females (n = 7) than males (n = 6) in comparison of surrounding and primary gliomas tissues, indicating that women patients may advance to high-grade glioma faster than men patients during early stage of tumor (Table 1, *p*<0.01). In contrast, while males (n = 16, Table 5) had higher frequency of promoter methylation, they underwent remarkable down-regulation of neogenin (n = 11, *p*<0.01) than females (n = 5, *p*>0.05) in recurrent gliomas (Table 4) implying that men patients may recur easily and progress faster in later stage of glioma. In most cases, larger tumors have worse pathological behaviour. Conformably, our data also showed that neogenin expression was negatively related to glioma size though this was not statistically significant. Meanwhile, the lowest neogenin level was detected in old aged patients who have been thought to experience a poor prognosis [5]. The average neogenin expression in the supratentorial cases was significantly lower than in subtentorial. Thus, it might mean that the malignancy of supratentorial group was more serious than subtentorial. In premise of increasing samples, the significant differences among glioma size, location and patient’s age might be clear.

Summarizing above, we suppose down-regulation of neogenin expression in gliomas is an important factor for glioma genesis, time to progression and recurrence. Survival time of the patients with lower neogenin might be shortened [31]. Thus neogenin may act a molecular marker for prognosis.

Promoter methylation is probably one of the reasons recognised to repress tumour suppressor genes in malignancy [34]. Our findings on neogenin methylation were in harmony with other dependent receptors such as DCC and UNC5H, where their loss of expression in several cancers was due to promoter methylation [44], [45]. In this study, the ratio of neogenin methylation increased with glioma grade possibly suggesting a strong positive correlation between promoter methylation and inactivation of neogenin. Considering that, we speculate promoter methylation to be one of the reasons for inactivation of neogenin expression in gliomas, high-grade gliomas in particular, resulting in insufficiency of neogenin at the cell membrane hence alter physiological functions working to the advantage of glioma genesis and/or progression. The observed methylation in gliomas reminisces the reports of existence of G-CIMP which was tightly associated with IDH1 mutation and exhibited better prognosis [35], [36]. Our observations in current study indicated neogenin did not belong to G-CIMP loci because of strong anti-correlation between neogenin methylation and G-CIMP-positive or IDH1 mutation status, similar to transmembrane protein tomoregulin (TMEFF2) which was hepermethylated and associated with worse prognosis [46]. Epigenetic alteration, however, cannot exactly explain all phenomena in our study. Perhaps other mechanisms like loss of heterozygosity or gene mutations which have been reported in DCC and Unc5H [47]–[49] could further provide more evidence to elaborate neogenin’s role in gliomas.

How does the deficiency of neogenin lead to tumorigenesis? The current view is that asymmetry of dependent receptors and their ligands would not only interrupt cell migration and differentiation but also actively inhibit the pro-apoptotic signalling [50]. Firstly, the ligands may be relatively surplus and free due to inactivation and downregulation of dependent receptors [26], [51]. Secondly, the ligands could gain expression in the malignant tumors [52]–[54]. If either or both of them happen, the surplus ligands would block apoptotic signal because of the mismatching of ligands and receptors [55], [56], thus the atypical glial cell would be immortalized to the advantage of tumorigenesis and perhaps accelerated. The mechanism fits for elaborating the observed dysfunction of neogenin in gliomas and induction of apoptosis in SHG-44 cell line after overexpression of neogenin further supports it. Another proposal is that lower neogenin expression could enhance angiogenesis in tumors [57]. It may be another factor to upgrade malignancy of glioma.

In summary, neogenin, one of the dependent receptors, is a tumor suppressor in glioma. Its expression is negatively correlated with glioma malignancy. And its loss even accelerates the process of glioma and recurrence after resection attributing to dysfunction of apoptosis. Methylated promoter of neogenin in gliomas may be one of the reasons for the downregulation of neogenin expression, indicating that adjustment of neogenin could be a strategy for glioma therapy.

## Materials and Methods

### Cell Culture and Transfection

Human glioma cell lines U251MG, U87MG and SHG-44 were obtained from the Cell Bank of Chinese Academy of Sciences (Shanghai, China), and were cultured in Dulbecco’s Modified Eagle Medium (DMEM), supplemented with 10% newborn calf serum at 37°C in an atmosphere of 5% CO_2_. SHG-44 cells were transiently transfected in 6-well plate with either human neogenin cDNA or the empty vector pcDNA3.1 using Lipofectamine 2000 reagent (Invitrogen).

### Glioma Tissue Samples

The fresh glioma tissues were obtained from the First Clinical Hospital of Jilin University, frozen in liquid nitrogen immediately after resection, including paired glioma and their surrounding tissues for Western blot analysis of neogenin expression (Table S1, n = 15), graded glioma tissues for methylation assay (Table S4, n = 33). The formalin fixed paraffin-embedded primary glioma sections (Table S2, n = 69 ) and paired initial and recurrent glioma samples (Table S3, n = 16) were from the Pathlogical Samples Library in the same hospital for immunohistochemical assay. All the sections were diagnosed by two independent neuropathologists to assign histological types and classified into 4 grades according to the 2007 WHO classification of tumors of the central nervous system. The fresh tissues were taken from the patients being in the hospital from January to November 2011. The primary patients who were in the hospital from January 2006 to January 2011 and the recurrent patients from January 2001 to January 2011 were treated with glioma resection only, without radiotherapy and chemotherapy. The tissues for immunohistochemistry were sectioned into 5 µm thick and re-checked by pathologists L. Q. and D. W. Permissions for using these materials were obtained from The Regional Ethical Review Board of Jilin University and Northeast Normal University, Changchun, China and written consents were obtained from all patients.

### Immunohistochemistry

Immunohistochemical analysis was performed on paraffin-embedded tissues sections. The antigen retrieval was performed by boiling sections in 10 mM citrate buffer pH 6.0 at the temperature of 100°C and the pressure of 0.12 MPa for 90 s. The immunohistochemical ultrasensitive™ S-P kit (Maixin Biotechnology, Fuzhou, China) was used for immunohistochemistry of neogenin. The procedure was in accordance with the instruction manual. Neogenin antibody was from Santa Cruz, USA. Chromogen reaction was performed according to DAB kit (Maixin Biotechnology, Fuzhou, China). At last, the sections were stained again with haematoxylin. The immunohistochemical sections were re-checked by independent pathologists L. Q. and Y. W.

### Western Blotting

The total protein of samples was extracted from normal brain tissues, paired glioma tissues and surrounding tissues. Equal amount of total protein from cell or tissue lysis in each lane was electrophoresed in 8% SDS-PAGE, transferred to PVDF membrane at the voltage of 70V for 2 hours. The goat anti-neogenin antibody (Santa Cruz Biotechnology, California, USA) was diluted as 1∶1000 to hybridize the neogenin in samples. The horse-radish peroxidase conjugated anti-goat IgG (1∶8000, Sigma-Aldrich, Missouri, USA) was used to recognise immunocomplexes which were finally visualised by ECL Plus Western blotting system (Amersham, Buckinghamshire, UK).

### Methylation-specific Polymerase Chain Reaction (MSP) [58]

EZ DNA Methylation- Direct™ Kit (the Epigenetics Company, California, USA) was used for methylation test. First genomic DNAs were extracted from tissues or cell lines. Then these DNAs were treated by bisulfite under the instruction manual. PCR conditions were set up as follows: 95°C initial Taq-Gold activation for 2 minutes, 95°C 30 seconds denaturation, 58°C 40 seconds annealing, 72°C 30 seconds extension for 35 cycles, and a final extension at 72°C for 5 minutes. The primer sequences were used as follows: the forward methylated primer 5′-CGCGTTAGGGTTTAGTAAGAGTC-3′, the reverse methylated primer 5′-ATATAAAACCTCAAAAAAAACCGAA-3′; the unmethylated forward primer 5′-TGTGTTAGGGTTTAGTAAGAGTTGG-3′, the unmethylated reverse primer 5′-ATATAAAACCTCAAAAAAAACCAAA-3′. Each MSP was repeated at least 3 times.

### Flow Cytometry

Cells were transfected with either empty vector or neogenin expression plasmid as previously described and harvested at 48 h. Subsequently, cells were stained with annexin V-FITC and propidium iodide for 15 min in dark. Flow cytometric analysis was performed according to instruction manual of Annexin V-FITC Apoptosis Detection Kit (KeyGEN Biotech, Nanjing, China).

### Statistical Analysis

All immunohistochemical pictures for calculating IOD were magnified 200 times and captured at the same pixel and the same white balance. Four pictures were taken in the random areas per section, which had no necrotic tissues. Positive signal IOD value which represents the neogenin level in pictures was calculated by the image pro-plus 6.0. SPSS18.0 was used to perform statistical analyses. One-Way ANOVA was used in the comparison of tumor grade, tumor size and patients’ age, while the comparison between the groups applied the Least Significant Difference method. Independent *t*-tests were used in the comparison of tumor latency, gender, length of being in hospital and location. Kaplan-Meier plot and Cox proportional hazards models were applied to analyse the associations between neogenin and high-grade glioma. The paired *t*-test was used in comparison of the primary and recurrent cases. With the *p*-value less than 0.05, the difference was statistically significant.

## Supporting Information

Table S1 **Clinicopathologic information and neogenin expression profile in paired surrounding and glioma tissues of 13 patients and 2 normal brains.** F: female; M: male; IOD: integral optical density; PD: pathological diagnosis; NB: normal brain tissue, NB1 is the normal tissue from a cerebral hemorrhage, NB2 is the tonsilla cerebelli from a Arnold-Chiari malformation patient; PA: pilocytic astrocytoma; LGA: diffuse astrocytoma; ODG: oligodendroglioma; MOA: oligoastrocytoma; AO: anaplastic oligodendroglioma; AA: anaplastic astrocytoma; AMOA: anaplastic mixed oligoastrocytoma; GBM: glioblastoma. The ratio is the value of neogenin divided by β-actin.
(PDF)

Table S2 **Clinicopathologic information and neogenin expression profile of 69 patients with primary glioma.** F: female; M: male; Sub: subtentorial; Sup: supratentorial; d: day; m: month; y: year; (o) represents the diameter recorded by visual inspection; IOD: integral optical density; PD: pathological diagnosis; PA: pilocytic astrocytoma; LGA: diffuse astrocytoma; ODG: oligodendroglioma; MOA: oligoastrocytoma; AO: anaplastic oligodendroglioma; AA: anaplastic astrocytoma.
(PDF)

Table S3 **Clinicopathologic information and neogenin expression profile of 16 primary and recurrent glioma patients.** F: female; M: male; IOD: integral optical density; PD: pathological diagnosis; LGA: diffuse astrocytoma; MOA: oligoastrocytoma; AO: anaplastic oligodendroglioma; AA: anaplastic astrocytoma; GBM: glioblastoma.
(PDF)

Table S4 **Clinicopathologic information of patients consisting of 29 gliomas and 4 non-neoplastic brain tissues who were checked by MSP.** MSP: methylation-specific polymerase chain reaction; NB: non-neoplastic brain tissues, NB1 is the normal tissue from a meningioma patient, NB2 is the normal tissue of a cerebral hemorrhage patient, NB3 and NB4 are the surrounding tissues of glioma; EO: ependymoma; PD: pathological diagnosis; PA: pilocytic astrocytoma; LGA: diffuse astrocytoma; ODG: oligodendroglioma; MOA: oligoastrocytoma; AO: anaplastic oligodendroglioma; GBM: glioblastoma; +: positive; −: negative.
(PDF)
