# Supplementary material for: Down-Regulation of Neogenin Accelerated Glioma Progression through Promoter Methylation and Its Overexpression in SHG-44 Induced Apoptosis
Source: PLoS One. 2012 May 29;7(5):e38074. doi: 10.1371/journal.pone.0038074 (PMC3362578; doi:10.1371/journal.pone.0038074)
Supplement: Table S1 — Clinicopathologic information and neogenin expression profile in paired surrounding and glioma tissues of 13 patients and 2 normal brains. F: female; M: male; IOD: integral optical density; PD: pathological diagnosis; NB: normal brain tissue, NB1 is the normal tissue from a cerebral hemorrhage, NB2 is the tonsilla cerebelli from a Arnold-Chiari malformation patient; PA: pilocytic astrocytoma; LGA: diffuse astrocytoma; ODG: oligodendroglioma; MOA: oligoastrocytoma; AO: anaplastic oligodendroglioma; AA: anaplastic astrocytoma; AMOA: anaplastic mixed oligoastrocytoma; GBM: glioblastoma. The ratio is the value of neogenin divided by β-actin. (PDF) [file pone.0038074.s001.pdf]

Table S1: Clinicopathologic information and neogenin expression profile in paired surrounding and glioma tissues of 13 patients and 2 normal brains (January - November, 2011).

ID	Gender	Age	PD	Grade	IOD of surrounding			IOD of glioma		
					neogenin	β-actin	Ratio	neogenin	β-actin	Ratio
1	M	41	NB1	—	2818.7	2884.4	0.977	—	—	—
2	M	47	ODG	II	161.9	3023.1	0.054	821	3731.4	0.22
3	M	56	GBM	IV	10264.2	3192.8	3.215	5465.4	3719.8	1.469
4	F	56	GBM	IV	660.9	2563.4	0.258	154.3	5617.4	0.027
5	F	25	LGA	II	9205	8656.7	1.063	5553.8	9601.5	0.578
6	M	58	AO	III	11305.3	7583.4	1.491	1398.1	5942.7	0.235
7	M	51	GBM	IV	3391.3	9269.5	0.366	973.9	4434	0.22
8	F	67	GBM	IV	9536.9	7930.7	1.203	461.1	8770	0.053
9	F	31	NB2	—	2612.6	9530.1	0.274	—	—	—
10	F	35	LGA	II	8522.9	6960.5	1.224	507.3	6775	0.075
11	M	49	AO	III	9049.6	11492.6	0.787	185.8	15008	0.012
12	M	38	MOA	II	8455.7	8147.5	1.038	6031.9	7246.1	0.832
13	F	40	PA	I	4873.8	10148.4	0.48	0	5039.1	0
14	F	48	GBM	IV	1809.4	2826.7	0.64	0	5602.1	0
15	F	30	MOA	II	1540.3	5777.1	0.267	283.1	7745.9	0.037

F: female; M: male; IOD: integral optical density; PD: pathological diagnosis; NB: normal brain tissue, NB1 is the normal tissue from a cerebral hemorrhage, NB2 is the tonsilla cerebelli from a Arnold-Chiari malformation patient; PA: pilocytic astrocytoma; LGA: diffuse astrocytoma; ODG: oligodendroglioma; MOA: oligoastrocytoma; AO: anaplastic oligodendroglioma; AA: anaplastic astrocytoma; AMOA: anaplastic mixed oligoastrocytoma; GBM: glioblastoma. The ratio is the value of neogenin divided by β-actin.
