# Supplementary material for: Down-Regulation of Neogenin Accelerated Glioma Progression through Promoter Methylation and Its Overexpression in SHG-44 Induced Apoptosis
Source: PLoS One. 2012 May 29;7(5):e38074. doi: 10.1371/journal.pone.0038074 (PMC3362578; doi:10.1371/journal.pone.0038074)
Supplement: Table S2 — Clinicopathologic information and neogenin expression profile of 69 patients with primary glioma. F: female; M: male; Sub: subtentorial; Sup: supratentorial; d: day; m: month; y: year; (o) represents the diameter recorded by visual inspection; IOD: integral optical density; PD: pathological diagnosis; PA: pilocytic astrocytoma; LGA: diffuse astrocytoma; ODG: oligodendroglioma; MOA: oligoastrocytoma; AO: anaplastic oligodendroglioma; AA: anaplastic astrocytoma. (PDF) [file pone.0038074.s002.pdf]

Table S2: Clinicopathologic information and neogenin expression profile of 69 patients with primary glioma (January, 2006 - January, 2011).

ID	Grade	Age/gender	PD	Location	Latency	In hospital (d)	Diameter (cm)	IOD
1	Ⅰ	23/F	PA	Sub	20d	24	2.5	13619.82
2	Ⅰ	15/M	PA	Sub	5y	20	5.72	15703.95
3	Ⅰ	23/M	PA	Sup	1m	20	3.6	12412.41
4	Ⅰ	4/F	PA	Sup	2.5y	18	6.9	2092.13
5	Ⅰ	30/M	PA	Sub	0.5y	19	2.2	11486.11
6	Ⅰ	18/F	PA	Sub	6m	15	3.2	1702.35
7	Ⅱ	16/M	LGA	Sub	6m	17	5.3	4243.96
8	Ⅱ	25/F	LGA	Sup	2y	19	3.5	11716.1
9	Ⅱ	61/M	LGA	Sup	0.5m	26	3	3672.71
10	Ⅱ	55/F	LGA	Sup	3y	17	4.83	5082.55
11	Ⅱ	38/M	LGA	Sup	3y	11	3(O)	6748.21
12	Ⅱ	43/M	LGA	Sup	10d	13	5.1	3080.02
13	Ⅱ	48/F	LGA	Sup	3y	30	5	18959.36
14	Ⅱ	56/M	LGA	Sub	45d	18	2.5	10723.56
15	Ⅱ	40/M	LGA	Sup	5y	33	6	15038.74
16	Ⅱ	4/M	LGA	Sup	1d	38	6.9	3886.12
17	Ⅱ	33/F	LGA	Sup	10m	28	1.78	6240.28
18	Ⅱ	32/M	LGA	Sup	2m	30	5(O)	1188.93
19	Ⅱ	8/M	LGA	Sup	1.5m	16	3.95	4001.6
20	Ⅱ	59/M	LGA	Sup	2y	19	2.5	2187.47
21	Ⅱ	29/M	ODG	Sup	6d	18	6(O)	11509.95
22	Ⅱ	30/F	MOA	Sup	1m	13	6	2613.97
23	Ⅱ	57/M	MOA	Sup	6m	15	4.4	4870.59
24	Ⅱ	48/M	MOA	Sup	1m	13	4(O)	999.5
25	Ⅱ	26/F	MOA	Sup	3m	17	5	4032.71
26	Ⅱ	21/M	MOA	Sup	4m	14	7.7	4757.26
27	Ⅱ	39/M	ODG	Sup	1m	14	5.58	6088.8
28	Ⅱ	33/F	MOA	Sup	6m	15	4.2	4490.62
29	Ⅱ	34/M	MOA	Sup	2m	11	5.8	14800.68
30	Ⅱ	56/M	MOA	Sup	2y	20	4(O)	10087.96
31	Ⅱ	54/F	LGA	Sup	2y	17	4.8	3964.74
32	Ⅱ	44/M	MOA	Sup	2y	14	8.1	15840.1
33	Ⅱ	15/F	LGA	Sup	1y	14	5.6	19181.37
34	Ⅱ	38/M	LGA	Sup	2m	13	4.93	6444.54
35	Ⅲ	55/M	AA	Sup	6m	14	4(O)	9334.13
36	Ⅲ	55/F	AA	Sup	1m	16	5(O)	1536.22
37	Ⅲ	55/F	AA	Sup	7d	16	4.43	1157.8
38	Ⅲ	40/F	AA	Sup	1m	19	4.8	1208.32
39	Ⅲ	57/F	AA	Sup	10y	16	5(O)	2983.17
40	Ⅲ	34/F	AO	Sup	7y	16	9.4	194.22
41	Ⅲ	73/F	AMOA	Sup	2M	22	8	448.78
42	Ⅲ	41/M	AMOA	Sup	4m	20	7	12821.2
43	Ⅲ	33/F	AMOA	Sup	1m	28	5.3	1151.08
44	Ⅲ	55/M	AA	Sup	20d	51	4.7	15034.03
45	Ⅲ	43/M	AA	Sup	7d	14	5	319.47
46	Ⅲ	28/M	AA	Sup	1w	14	4.5	48.43
47	Ⅲ	48/F	AA	Sup	2y	16	4	744.12
48	Ⅲ	48/F	AA	Sup	1y	19	2.7	3785.6
49	Ⅳ	65/F	GBM	Sup	7d	20	9.88	7137.72
50	Ⅳ	14/M	GBM	Sup	15d	62	6	4827.78
51	Ⅳ	37/M	GBM	Sup	1y	14	5.04	6166.14
52	Ⅳ	51/M	GBM	Sup	1.5m	13	4.24	4633.47
53	Ⅳ	51/M	GBM	Sup	2y	13	3.6	14759.33
54	Ⅳ	59/F	GBM	Sup	20d	14	4	787.74
55	Ⅳ	41/F	GBM	Sup	20d	12	5.43	980.07
56	Ⅳ	27/F	GBM	Sup	3m	18	5.5(O)	116.94
57	Ⅳ	49/F	GBM	Sup	1m	16	2.78	864.7
58	Ⅳ	53/M	GBM	Sup	10d	18	3.26	1256.67
59	Ⅳ	63/M	GBM	Sup	10m	21	6.67	2054.52
60	Ⅳ	25/F	GBM	Sup	1m	21	5.15	2041.19
61	Ⅳ	76/M	GBM	Sup	1y	19	5.6	1092.14
62	Ⅳ	20/F	GBM	Sup	1m	13	5.8	2261.09
63	Ⅳ	12/M	GBM	Sup	20d	17	3(O)	609.55

Table S2 (Wu et al.)

64	Ⅳ	55/F	GBM	Sup	2y	22	3(O)	8267.42
65	Ⅳ	54/M	GBM	Sup	7d	24	6(O)	520.59
66	Ⅳ	45/F	GBM	Sup	1m	18	3.8	795.79
67	Ⅳ	52/M	GBM	Sup	1w	28	6	2797.73
68	Ⅳ	52/F	GBM	Sup	0.5m	14	4.6	477.8
69	Ⅳ	55/F	GBM	Sup	10d	14	4	7890.92

F: female; M: male; Sub: subtentorial; Sup: supratentorial; d: day; m: month; y: year; (o) represents the diameter recorded by visual inspection; IOD: integral optical density; PD: Pathological diagnosis; PA: pilocytic astrocytoma; LGA: diffuse astrocytoma; ODG: oligodendroglioma; MOA: oligoastrocytoma; AO: anaplastic oligodendroglioma; AA: anaplastic astrocytoma.
