# Supplementary material for: Down-Regulation of Neogenin Accelerated Glioma Progression through Promoter Methylation and Its Overexpression in SHG-44 Induced Apoptosis
Source: PLoS One. 2012 May 29;7(5):e38074. doi: 10.1371/journal.pone.0038074 (PMC3362578; doi:10.1371/journal.pone.0038074)
Supplement: Table S3 — Clinicopathologic information and neogenin expression profile of 16 primary and recurrent glioma patients. F: female; M: male; IOD: integral optical density; PD: pathological diagnosis; LGA: diffuse astrocytoma; MOA: oligoastrocytoma; AO: anaplastic oligodendroglioma; AA: anaplastic astrocytoma; GBM: glioblastoma. (PDF) [file pone.0038074.s003.pdf]

Table S3: Clinicopathologic information and neogenin expression profile of 16 primary and recurrent glioma patients (January, 2001 - January, 2011).

		Primary				Recurrent			
ID	Sex	Age	PD	Grade	IOD	Age	PD	Grade	IOD
1	M	55	AA	Ⅲ	15034.03	55	AA	Ⅲ	959.3
2	F	54	LGA	Ⅱ	3964.74	54	AA	Ⅲ	1466.65
3	M	52	GBM	Ⅳ	2797.73	55	LGA	Ⅱ	7348.74
4	M	39	AC	Ⅱ-Ⅲ	40046.46	40	AC	Ⅱ-Ⅲ	3616.15
5	M	28	AA	Ⅲ	48.43	30	AC	Ⅲ-Ⅳ	552.82
6	F	52	GBM	Ⅳ	477.8	52	GBM	Ⅳ	638.91
7	M	51	LGA	Ⅱ	21552.02	52	LGA	Ⅱ	5982.32
8	F	48	AA	Ⅲ	744.12	50	GBM	Ⅳ	10954.46
9	M	44	GBM	Ⅳ	22548.43	45	GBM	Ⅳ	477.16
10	F	48	AA	Ⅲ	3785.6	50	GBM	Ⅳ	6061.18
11	M	44	MOA	Ⅱ	15840.1	46	MOA	Ⅱ	3351.96
12	F	15	LGA	Ⅱ	19181.37	18	AO	Ⅲ	886.46
13	M	53	AC	Ⅱ-Ⅲ	4587.95	53	LGA	Ⅱ	5927.71
14	M	38	LGA	Ⅱ	6444.54	39	GBM	Ⅳ	1745.52
15	M	37	AC	Ⅲ-Ⅳ	49159.17	38	GBM	Ⅳ	12283.68
16	M	55	GBM	Ⅳ	7890.92	56	GBM	Ⅳ	4136.14

F: female; M: male; IOD: integral optical density; PD: pathological diagnosis; LGA: diffuse astrocytoma; MOA: oligoastrocytoma; AO: anaplastic oligodendroglioma; AA: anaplastic astrocytoma; GBM: glioblastoma.
