# Supplementary material for: Down-Regulation of Neogenin Accelerated Glioma Progression through Promoter Methylation and Its Overexpression in SHG-44 Induced Apoptosis
Source: PLoS One. 2012 May 29;7(5):e38074. doi: 10.1371/journal.pone.0038074 (PMC3362578; doi:10.1371/journal.pone.0038074)
Supplement: Table S4 — Clinicopathologic information of patients consisting of 29 gliomas and 4 non-neoplastic brain tissues who were checked by MSP. MSP: methylation-specific polymerase chain reaction; NB: non-neoplastic brain tissues, NB1 is the normal tissue from a meningioma patient, NB2 is the normal tissue of a cerebral hemorrhage patient, NB3 and NB4 are the surrounding tissues of glioma; EO: ependymoma; PD: pathological diagnosis; PA: pilocytic astrocytoma; LGA: diffuse astrocytoma; ODG: oligodendroglioma; MOA: oligoastrocytoma; AO: anaplastic oligodendroglioma; GBM: glioblastoma; +: positive; −: negative. (PDF) [file pone.0038074.s004.pdf]

Table S4: Clinicopathologic information of patients consisting of 29 gliomas and 4 non-neoplastic brain tissues who were checked by MSP (January - November, 2011).

ID	Gender	Age	PD	Grade	Methylated	Unmethylated
1	F	48	NB1	—	-	+
2	M	41	NB2	—	-	+
3	F	40	NB3	—	-	+
4	M	58	NB4	—	-	+
5	F	40	PA	I	-	+
6	M	26	PA	I	-	+
7	M	47	ODG	II	-	+
8	F	50	ODG	II	-	+
9	M	38	MOA	II	-	+
10	F	15	LGA	II	+	+
11	F	30	MOA	II	-	+
12	F	25	LGA	II	-	+
13	M	17	EO	II	-	+
14	M	26	LGA	II	-	+
15	M	58	AO	III	+	+
16	M	62	AO	III	+	+
17	F	56	GBM	IV	-	+
18	F	61	GBM	IV	-	+
19	M	59	GBM	IV	+	+
20	M	62	GBM	IV	-	+
21	F	67	GBM	IV	+	+
22	M	64	GBM	IV	+	+
23	F	26	GBM	IV	-	+
24	M	49	GBM	IV	+	+
25	M	51	GBM	IV	-	+
26	F	48	GBM	IV	-	+
27	M	56	GBM	IV	+	+
28	F	45	GBM	IV	-	+
29	F	56	GBM	IV	-	+
30	F	58	GBM	IV	-	+
31	M	49	GBM	IV	-	+
32	F	61	GBM	IV	-	+
33	F	47	GBM	IV	+	+

MSP: Methylation-specific polymerase chain reaction; F: female; M: male; NB: non-neoplastic brain tissues, NB1 is the normal tissue from a meningioma patient, NB2 is the normal tissue of a cerebral hemorrhage patient, NB3 and NB4 are the surrounding tissues of glioma; EO: ependymoma; PD: pathological diagnosis; PA: pilocytic astrocytoma; LGA: diffuse astrocytoma; ODG: oligodendroglioma; MOA: oligoastrocytoma; AO: anaplastic oligodendroglioma; GBM:

glioblastoma; +: positive; -: negative.
